# Continuity of care: trust-based relationship and availability of personalized information in user experience

**DOI:** 10.1590/0102-311XEN109524

**Published:** 2025-07-18

**Authors:** Patty Fidelis de Almeida, Adriano Maia dos Santos, Rafaela Fidelis Lima Silvério, Amanda Maria Villas Bôas Ribeiro, Drieli Oliveira Silva, Ana Luiza Queiroz Vilasbôas

**Affiliations:** 1 Instituto de Saúde Coletiva, Universidade Federal Fluminense, Niterói, Brasil.; 2 Instituto Multidisciplinar em Saúde, Universidade Federal da Bahia, Vitória da Conquista, Brasil.; 3 Instituto de Saúde Coletiva, Universidade Federal da Bahia, Salvador, Brasil.

**Keywords:** Continuity of Patient Care, Integrality in Health, Health Services Accessibility, Continuidad de la Atención al Paciente, Integralidad en Salud, Accesibilidad a los Servicios de Salud

## Abstract

This article analyzes the continuity of care in the relational domain based on user experience. This is a qualitative case study based on 45 interviews with person living with HIV (PLH) followed-up in polyclinics and 38 interviews with users diagnosed with systemic arterial hypertension (SAH) registered in basic health units (BHU) in a large city in the state of Rio de Janeiro, Brazil. The results were analyzed according to two dimensions of relational continuity: trust-based relationship and availability of personalized information. The strongest trust-based relationships and the protagonism in the availability of health information were established with the family health team (FHT) physician and the infectious disease specialist in the polyclinics. Among the differences, according to users with SAH, the relationship bonds were broken by health care provider turnover. In the absence of relationships with other FHT professionals, continuity and access were simultaneously affected. Among PLH users, nursing professionals played an important role in care. In both cases, the results showed a care with little concern for interprofessional practices, collective actions and promotional initiatives to strengthen continuity. The lack of technology-mediated communication required users to attend health care services to solve their demands and contact professionals. The results indicate the importance of a regular point of care for establishment of the bond and consequent relational continuity. However, health care work management-related problems in the Brazilian Unified National Health System aggravate health care provider turnover and favor disruptions in therapeutic follow-up.

## Resumen

En este artículo se analiza la continuidad de la atención en el ámbito relacional desde la experiencia del usuario. Se llevó a cabo un estudio de caso cualitativo basado en 45 entrevistas con personas que viven con VIH (PVH) monitoreadas en policlínicas y 38 con usuarios diagnosticados con hipertensión arterial sistémica (HAS) registrados en unidades básicas de salud (UBS) en un gran municipio del estado de Rio de Janeiro, Brasil. Los resultados se analizan desde dos dimensiones de la continuidad relacional: relaciones de confianza y disponibilidad de información personalizada. Junto con el médico del equipo de salud familiar (EqSF) y el infectólogo en las policlínicas, se establecieron las relaciones de confianza más fuertes y el protagonismo en la disponibilidad de información de salud. Entre las diferencias para los usuarios con HAS, los bonos se dividieron en función de la rotación médica. En ausencia de relaciones con otros profesionales de EqSF, la continuidad y el acceso se vieron afectados simultáneamente. Entre las PVH, los profesionales de enfermería desempeñaron un papel importante en la acogida. En ambos casos, los resultados revelaron poco cuidado con las prácticas interprofesionales, acciones colectivas y promocionales para fortalecer la continuidad. La ausencia de comunicación mediada por tecnologías requiere que los usuarios acudan a los servicios para resolver sus demandas y contactar con los profesionales. Los resultados revelan la importancia de un punto de atención regular para lograr el vínculo y la consecuente continuidad relacional. Por otro lado, los problemas relacionados con la gestión laboral en el Sistema Único de Salud intensifican la rotación profesional y favorecen las rupturas en el seguimiento terapéutico.

## Introduction

Continuity of care has been associated with reduced hospitalizations, mortality and use of emergency services, in addition to strengthening adherence to health promotion and the relationship between professionals and users ^1^ ^,^ ^2^ ^,^ ^3^ ^,^ ^4^. Its achievement represents a major challenge to health care systems worldwide, whose implementation of policies and actions is concentrated in the scope of primary health care (PHC) ^4^ ^,^ ^5^. However, for people living with chronic diseases or complex health needs, ensuring continuity involves specialized, hospital, social and community services ^6^ ^,^ ^7^.

The classic concept by Haggerty et al. ^8^, widely adopted and adapted to different contexts, proposes a framework of continuity of care in three domains: informational, managerial and relational. The first presupposes the continuous use of relevant information about the patient’s history and their living and health conditions with a view to making current and future care adequate. Managerial continuity involves coherence in clinical management based on cooperation and communication between the various providers to provide care in a timely manner and without duplication. The domain of relational continuity, the focus of this article, involves the quality and coherence of the relationship produced between health care professionals/team and users ^8^ ^,^ ^9^.

The interdependence between the three domains is undeniable. It is argued that, although continuity is a multidimensional construct, it needs to be considered in the process of organizing health care services and, above all, in user experience ^10^. For users, family members and caregivers, continuity is perceived and facilitated when they receive support from professionals or teams that assume responsibility for therapeutic plans and offer support in the management of various resources (medicines, treatments, functioning of the health system) ^11^.

Relational continuity is positioned in the micro dimension of the health care system. It implies the establishment of lasting and personalized relationships between professionals/teams and users, as well as a holistic approach to health problems ^4^. It represents more than “having contact with the same doctor”, but rather a 2-way commitment based on mutual trust and the health care provider’s responsibility for their patients, whose intensity increases as the relationships become more stable and sustainable ^10^. From this perspective, continuity is close to the concept of “longitudinality” by incorporating the establishment of trust-based relationships over time with a provider recognized as a regular source of care ^12^.

Sidaway-Lee et al. ^3^ note that relational continuity has effects on health outcomes through mechanisms such as frequent contact, accumulated knowledge, sense of responsibility of the professional, quality of the relationship, trust, and empathy. Maintaining the same health care team favors such continuity, enabling relationships based on user needs (clinical, mental, social and environmental needs) and adequate access to available resources ^13^. Thus, it favors adherence to and trust in the guidance of professionals ^11^, who, in turn, with prolonged contact, develop a more comprehensive view of the care offered in the various health care services and the role of family members ^4^.

In Brazil, social inequality and difficulties in the operationalization of Health Care Networks (HCN) have effects on the continuity of care, which often depends on informal mechanisms adopted by users and family members ^14^. Brazilian studies ^12^ ^,^ ^15^ ^,^ ^16^ indicate weaknesses in the interpersonal relationship between users and professionals, possibly related to difficulties in accessing PHC. Even so, a set of strategies and tools for continuity of care were found, such as safe discharge, clinic management, case discussion, lines of care, case manager nurse, regulatory complexes and permanent education, although not necessarily implemented in the country ^17^. However, organizational arrangements that catalyze work processes in an interprofessional perspective for effective relational continuity and shared communicational flow remain little explored, especially in situations of health care gaps ^18^, conflicted territories ^19^ and for populations whose approach to health care requires higher cultural competence ^20^.

This article analyzes the continuity of care in the relational domain based on the experience of person living with HIV (PLH) whose care is centralized in a specialized service and users with systemic arterial hypertension (SAH) registered in basic health units (BHU). We seek to answer the following research question: considering different health care contexts (specialized services and BHUs), what factors favor or hinder relational continuity from the users’ perspective? Both conditions, due to their chronic nature, require continuous follow-up that contributes to adherence to individual and collective health care. It is argued that, despite technical advances, especially in biomedical technologies, relational factors continue to be important predictors of health outcomes even though, contradictorily, there is a decrease in the value attributed to personal contact between teams, professionals and users ^1^. In addition, comparing diversified experiences in different types of health care providers and conditions deepens the understanding of the continuity of care, which is one of the main contributions of this study. Accordingly, most analysis models and concepts are based on studies in high-income countries, contrasting with the reality of contexts with limited resources and high fragmentation, such as that of Brazil ^14^ ^,^ ^21^.

## Methodology

### Study type

This is a case study, with a qualitative approach, based on interviews with users diagnosed with SAH, registered by Family Health teams (FHTs), and PLH users followed-up in polyclinics. The participants’ care trajectories in the search for PHC health care to the other points of the HCN were reconstructed, including in the private sector. For this article, we traced and analyzed results that informed on the relational domain of continuity of care.

### Study setting and participant selection criteria

The study setting was a municipality located in the state of Rio de Janeiro, Brazil, with a population of approximately 500,000 inhabitants.

We conducted 38 face-to-face interviews with users with SAH, from February to July 2022, in 10 of the 43 BHUs; and 45 interviews with PLH, between December 2021 and June 2022, in the seven municipal specialty polyclinics that, in the study setting, are the main follow-up services.

The selection prioritized BHUs with 3 to 6 FHTs, distributed in the seven health regions, due to the COVID-19 pandemic, which reduced the flow of users, making it difficult to find them in services with fewer teams. In addition, the selection of BHUs with this profile considered the vacancy/exchange of professionals, resulting from a public service admission exam during the study period.

After selection of BHUs, possible participants were identified by the interviewers with the help of the respective FHT, according to the inclusion criteria: having a diagnosis of SAH and being registered by an FHT; request for referral by the PHC for consultation or specialized examination in the last 12 months prior to the interview (verified in the medical records or registration books); being 18 years old or older; and not presenting physical or psychological impairment impeding the interview, according to the evaluation of the FHT professionals. Based on this initial selection, which varied in each of the selected BHUs, potential participants were invited to attend the service or consulted about the possibility of conducting the interview at home.

Regarding PLH users, users linked to the seven municipal polyclinics were interviewed, based on the inclusion criteria: being 18 years old or older; residing in the municipality; with positive HIV testing for at least one year prior to the interview. Participants were identified, with the help of service professionals, and invited to participate during the wait or shortly after the consultation with the infectious disease specialist.

### Data production

The interviews were conducted by previously trained researchers and adopted a semi-structured guide. Regarding the two health problems, the interview guide contained the following blocks: sociodemographic profile, habits and lifestyle, diagnosis discovery, care received in PHC and specialized care, other paths taken in the Brazilian Unified National Health System (SUS, acronym in Portuguese) and in the private network, care during the COVID-19 pandemic and general assessment of the care received. Continuity of care, as understood in this article, is the “amalgam” that favors the users’ perception of the care continuum and was addressed within the experiences of care at each health care level or service and in the transition between them. Interviews with SAH users, with an average of 30 minutes, and interviews with PLH, with an average of one hour, were recorded, transcribed in full for analysis and interpretation of the results. Data production was terminated based on the obtention of adequate and sufficient information to understand the phenomenon ^22^, composed of accounts of users of the seven health care areas of the municipality.

### Data analysis

For the analysis of the results, general ordering of data was carried out. The transcripts were read exhaustively and the thematic nuclei were categorized based on the pre-established dimensions for relational continuity. The interpretation was guided by thematic content analysis, identifying convergences and divergences ^23^. In addition, the most representative excerpts were selected and distributed in the respective categories.

For the description and interpretation of the results, based on the reference of Haggerty et al. ^8^ and complementary references ^3^ ^,^ ^4^ ^,^ ^10^ ^,^ ^11^ ^,^ ^21^ ^,^ ^24^, dimensions linked to the domain of relational continuity - trust-based relationships and availability of personalized information - and their respective definitions were previously identified, presented in Box 1. Subsequently, the reports produced through the interviews were compared to the dimensions to define and adapt the actions/activities that compose the analysis matrix (Box 1).

**Box 1:** Dimensions, definition and actions/activities of relational continuity in user experience.

DIMENSIONS	DEFINITION	USER-PERCEIVED ACTIONS/ACTIVITIES
Trust-based relationship between health care providers/services and users	It presupposes the establishment of stable and personalized therapeutic relationships between health care providers/teams and users and a holistic approach to health problems that considers their resources, preferences and needs. It involves the team/professional responsibility for users. From this perspective, trust influences user behavior in better adherence to guidelines, medications and preventive measures	Identification of a reference professional/coordinator of care Perception that the health care professional/team has a holistic view of their clinical/personal history Trust in the health care professional/team Singular therapeutic project that guides the conduct of primary health care team professionals and others involved
Availability of personalized information	It refers to communication with exchange of clear information about the health condition, adapted to the unique abilities and needs of each user, respecting even the individual times to deal with situations of illness/treatment. It includes considering each user's prior knowledge or understanding about their condition	Receiving clear, understandable and personalized guidance Confidence for clarification of doubts and exchange of information Knowledge co-production in the development of health literacy and self-care Availability of devices to receive information, appointment reminders, among others Access to contacts/phone numbers of health care providers/services involved in the care

Source: based on Haggerty et al. ^8^; Ljungholm et al. ^4^ ^,^ ^11^; Nóbrega et al. ^24^; Hill et al. ^10^; Meiqari et al. ^21^; Sidaway-Lee et al. ^3^.

The proposed model, although it seeks to identify elements related to the domain of relational continuity, should be considered in the light of the relation between the other components - informational and managerial, since they are dependent and interrelated ^18^.

The study was approved by the Research Ethics Committee of the Human Sciences Institute , Fluminense Federal University (opinion n. 4,456,756), with the consent of the municipality. Participants were identified by acronym (PLH or SAH), number that corresponds to the interview and a letter assigned to each service - polyclinic in the case of HIV and BHU in the case of SAH.

## Results

### Respondent profile

Table 1 shows the profile of the study participants. Among the PLH, about half were men, the age group was concentrated between 41 and 60 years, with partners and children. The vast majority identified themselves as black and mixed-race (75.5%), with high school education (44.4%), family income of up to two monthly minimum wages (53.3%), and as home providers (53.3%). Among the 38 participants diagnosed with SAH, there was a predominance of females (76.3%), self-declared black and mixed-race (71%) and aged over 60 years (55.3%). Most of them were the main providers of the household (60.5%), with a family income of up to one monthly minimum wage (52.6%). Half of the participants with SAH (50%) received some type of social or social security benefit. In both groups, most did not have health insurance plans, especially among users with SAH, who also had lower income (Table 1).

**Table 1:** Characterization of study participants. Large municipality, Rio de Janeiro, Brazil, 2022.

Socioeconomic characteristics	PLH		SAH	
	n	%	n	%
Age (years)				
< 49	29	64.5	6	15.7
50-59	10	22.2	11	28.4
60-69	4	8.9	13	34.2
70-79	2	4.4	7	18.4
80 or more	0	0.0	1	2.6
Race/Color (self-declared)				
White	8	17.8	11	28.4
Mixed-race	22	48.9	18	47.3
Black	12	26.7	9	23.6
Indigenous	0	0.0	0	-
Asian	2	4.4	0	-
I prefer not to answer	1	2.2	-	
Gender				
Cis man	23	51.1	8	21.1
Cis woman	18	40.0	29	76.3
*Travesti*	1	2.2	1	2.6
Non-binary	2	4.4	-	-
I prefer not to answer	1	2.2	-	-
Schooling				
Incomplete elementary school	3	6.7	11	28.4
Complete elementary school	3	6.7	11	28.4
Incomplete high school	7	15.6	5	13.2
Complete high school	20	44.4	10	26.3
Incomplete higher education	3	6.7	1	2.6
Complete higher education	9	20.0	-	-
Family income (minimum wage)				
< 1	-	-	20	52.6
1-2	23	51.1	10	26.3
Up to 3	11	24.4	5	13.2
Up to 4	7	15.6	2	5.3
More than 4	2	4.4	-	-
Declares having no income	1	2.2	1	2.6
Did not know	1	2.2	-	-
Social benefit *				
No	30	66.7	19	50.0
Yes	15	33.3	19	50.0
Retirement/Pension	5	-	7	36.8
Continuous Cash Benefit	0	-	1	5.3
Municipal Social Benefit	3	-	7	36.8
Brazil/Emergency Aid	7	-	4	21.1
*Bolsa Família*	6	-	-	-
Health insurance plan				
None	28	62.2	31	81.6
Health insurance plan	6	13.3	2	5.3
Low-cost health insurance plan	11	24.5	5	13.1

PLH: person living with HIV; SAH: users with systemic arterial hypertension.

Source: prepared by the author based on the interviews.

* Some PLHs reported receiving more than one benefit; therefore, it was not possible to calculate the percentage of the specific benefits.

### Trust-based relationship between health care providers/services and users

The FHT physician was the main reference for the follow-up of users with SAH over time, although due to the public selection that occurred at the time and the post-COVID-19 pandemic context, several professionals were no longer part of the teams. Users resented the changes and explained that “*Here* [BHU] *there is no continuity for anything*” (SAH/30/H). A significant set of participants reinforced the deleterious effects of health care provider turnover on the rupture of bonds and the abandonment of the search for the health care service (Box 2). In the same context of complaints about provider turnover, spontaneous accounts about the relationship established with Cuban doctors from the More Doctors Program who worked in some BHUs, until 2018, were recorded. Aspects defined as “kindness”, attention and integral view of the subject were related to satisfaction and the feeling of being cared for (Box 2).

**Box 2:** Trust-based relationship between health care providers/services and users - expressive accounts of participants. Large municipality, Rio de Janeiro, Brazil, 2022.

ACTIONS/ACTIVITIES	EXPRESSIVE ACCOUNTS	
	PLH	SAH
Identification of a reference professional/coordinator of care	“*Anything I feel, I go talk with her* [infectious disease specialist] *first* [...] *My life* [laughs]*. They coordinate my life...*” (PLH/16/D) “*...I suffered a lot. Because from the time I was 9 until I was 16 or 17, there were always the same people, and when I saw them leave, that’s when I think everything about my life started to unravel, for the treatment I mean. Because I was very attached to the psychologist, to the doctor, to the social worker, and the nurses. Then, when they left, I suffered. It wasn’t the same thing anymore. I’d go to the appointment untidy, like, I’d go just for the sake of it. Because before, when they were there, I made a point of going all dressed up to see them*” (PLH/5/A) “[The CHW] *is my neighbor, but I have no contact with her, none. When I needed it, I avoided it, because I also didn’t want the entire street knowing that I had HIV, especially after I broke up. It would be awful for me*” (PLH/45/A)	“*Now I haven’t even been going. There is no doctor at the clinic... They would stay for two, three months, then quit. It would be about two months without a doctor, three months. Like it is now, there is no doctor. It only serves us when we get there very ill*” (SAH/20/C) “*Then there was a lot of change of staff, there was a time that... like, it was... then I started checking it at home. I said, like: ‘look, if it’s above 14, I’ll go to the doctor’*” (SAH/36/J) “*Yeah, that Cuban doctor. Oh, I loved her! She was really good, you know? She served you, we didn’t understand much of her talk, but she served us with a total kindness.* [...] *And she would see me every three months...*” (SAH/31/H). “*She was good because: ‘Look, I’m not going to treat your disease, I’m going to treat you.’ I loved when she said that... Then, she examined me from inside out*” (SAH/30/H) “[name]*, I can’t right now...’, then he* [CHW] *would schedule an appointment with the doctor* [name] *and I would come. ‘Oh, come that I’ll schedule it.’ He was great. Now he's not here anymore...*” (SAH/4/A) “*I say: that’s how it is going, like this, in such a way’ ‘*[nursing technician] *I’ll help you with that!’ So it’s a person that we have more access to, you know? If you need help, go to the facility and look for her* [nursing technician name]” (SAH/13/D)
Perception that the health care professional/team has a holistic view of their clinical/personal history	“*I think she* [infectious disease specialist] *is the person who knows the most about me. Just like the Big Brother TV show, people go to the Big Brother to get to know one another. And she’s the one who knows the most about me*” (PLH/37/C) “*The ones I trust the most are both the doctor* [infectious disease specialist name] *and this nurse who came here, because he is also always following closely, asking if everything is okay, if you need any help, how much medicine I have left, if I need more of it*” (PLH/15/D) “*Yeah, I’m very fond of them* [nursing staff]*. In fact, it’s more like gratitude for what they do. They help us a lot. So, we don’t just have medical support, you know? It’s an emotional support that we also need. That’s why I like it here*” (PLH/1/B)	“*Now everything is new, everything has changed, I’m not familiar with them, I don’t know anyone because I almost don’t come here*” (SAH/8/B) “*But that’s it, they are different people who do not have that daily life like us. The previous doctors are old doctors who knew us. Nowadays, doctors don’t know the patients here. So it’s very complicated to talk about some issues*” (SAH/2/A)
Trust in the health care professional/team	“*I have focused my care a lot on her* [infectious disease specialist]*. I left my life in her hands, so she always knows what I need, what I don’t need, she always pays attention to every little detail*” (PLH/15/F) “*Everything, blood test, medicine, appointment, other types of illness, fever, everything with her. She is the doctor I trust the most*” (PLH/36/C)	“*Currently, I don’t come here* [BHU]*. Everything has changed...* [...] *You know, I can’t explain it to you?* [laughs]*. I don’t know if it was more attention, there was more attention.* [...] *Like, more trust, then there was more: ‘you can’t stop coming’, then* [the doctor] *advised: ‘Stop smoking, quit the cigarettes...’*” (SAH/8/B)
Singular therapeutic project that guides the conduct of PHC team professionals and others involved	Question: “*And the doctor there* [PHC]*, does he make any referral, any report for you to give the doctor here* [Polyclinic]*?*” Answer: “*No*” Question: “*And the doctor* [infectious disease specialist] *does she make some to give there?*” Answer: “*No. Here is here and there is there*” Question: “*Do the doctors talk to each other?*” Answer: “*No*” (PLH/27/B) “*This could have already improved in that sense. The doctor wanted to know my entire HIV history so then he would know how he was going to treat me. And I was there dying of pain* [in an emergency service]” (PLH/42/E)	“*If I’m not under a treatment plan, then I might get a really terrible health condition. Which is what was happening. I was always going to the hospital with high blood pressure and never being able to have the monitoring here* [BHU]” (SAH/11/C). “*I think that if there were an exchange, a good internet connection, a good work, when I got here with my medical record number in that specialty, everything should already be prescribed in it. You have to repeat it all over again! These are things that... I’m not smart enough, I didn’t go to college, but I know what bothers me. You know?*” (SAH/11/C)

BHU: basic health unit; CHW: community health worker; PHC: primary health care; PLH: person living with HIV; SAH: users with systemic arterial hypertension.

Source: prepared by the authors based on the interviews.

Unequivocally, PLH indicated the infectious disease specialist as the main reference for monitoring health in general, and, in some cases, the only one. They highlighted the strength of the bond with the infectious disease doctor, recognized as the professional to whom “*life was entrusted*” (PLH/15/F). Mention was made of the care provided by nurses, in four of the seven services. The regularity in the pre-scheduling of appointments with the infectious disease specialist - quarterly or biannual - was pointed out by the PLH as favoring the bond and access. Most respondents considered themselves assiduous in appointments and recognized that, mainly, infectious disease specialists and nurses had an expanded understanding of their clinical and life histories. Although, at the time, there was also public selection for polyclinics professionals, turnover did not appear in an important way in the accounts, although some users were afraid of possible changes of professionals based on previous experiences (Box 2).

Community health workers (CHWs) were mentioned by users with SAH as an important reference for the intermediation of the demands presented to the BHU, communicating about the possibility of scheduling appointments, status of appointments for specialized services, results of exams, absence of professionals and updating of the registration (Box 2). In the case of PLH, there were no reports of bonds with CHWs. Just over half of the participants (26 PLH) reported having a BHU registration and only four reported using it regularly. When home visits occurred, they were commonly directed to family members with health problems and, rarely, to PLH. The activities reported were similar to those of users with SAH, but directed to other family members. In addition, the PLH pointed out the fear about the breach of diagnostic confidentiality as an obstacle to bonding with the CHWs (Box 2).

Subsequently, several participants with SAH cited nursing technicians as a reference, especially for procedures such as blood pressure measurement, anthropometry, capillary glycemia and care in BHUs. The nurse was referred to in a few cases, and clinical appointments for the management of SAH were not reported. Among the PLH, the nursing professional, in some polyclinics, had a role highlighted and valued by the participants in care and active search (telephone/message) in cases of absenteeism. These professionals organized the flow of internal care in the service, since some of these units had no fixed offices for appointments and users felt lost and exposed (Box 2).

Participants with SAH emphasized that clinical histories were lost due to physician turnover, which also broke the experience of “intimacy” (Box 2). In addition to the rupture of bonds, vacancy periods aggravated problems of access to PHC. The monitoring of SAH was restricted, and attempts to “fitting into schedule”, through spontaneous demand, were common. Several SAH users gave up seeking the BHU or sought other forms of care such as measuring blood pressure at home or seeking appointments and exams in low-cost health insurance plans (Box 2).

In the case of PLH, turnover did not appear significantly. Participants considered themselves linked to polyclinics and sought professionals for almost all health problems. There were very frequent reports of seeking an infectious disease specialist in situations such as influenza, hypertension control, among other needs. Several users presented to the infectious disease doctor prescriptions and results of tests prescribed by other professionals - from PHC, hospitals, emergency care units or specialists from the private network - for follow-up purposes or even for conduct review (Box 2).

SAH and PLH participants reported no strategies or actions that could constitute the construction of a singular therapeutic project (STP) between the FHT and polyclinics, with the participation of other health care providers or services. In polyclinics, despite the possibility of internal referrals to other specialists, there were no reports of STP production. The delegation of the function of communicating the respective clinical and life histories was under the responsibility of the users or family members, which required memory, understanding of all guidelines and prescriptions to transmit them in the various appointments, which generated dissatisfaction (Box 2).

### Availability of personalized information

The primary source of information for SAH care was the physician. Some reports indicated that the trust necessary to clarify doubts or exchange information about health problems was established only with this professional. For PLH, infectious disease specialists and nurses played this role (Box 3).

**Box 3:** Personalized information - expressive accounts of participants. Large municipality, Rio de Janeiro, Brazil, 2022.

CATEGORIES	EXPRESSIVE ACCOUNTS	
	PLH	SAH
Receiving clear, understandable and personalized guidance	“*And she* [infectious disease specialist] *explains to me the reason of everything: ‘that’s why I’m withdrawing this medication and introducing this medication...’*” (PLH/18/C) “*What a pity! I can’t because in public health it is very difficult, there are a lot of people, a huge queue of people to serve. The doctor, if she stays 40 minutes with me, talking to me, it’s too much. There are a lot of stressed people waiting outside*” (PLH/17/C)	“*With the doctors, yeah, for sure. The other professionals, like, not so much... They know* [other professionals] *that I have diabetes, that I have high blood pressure, but that I will expose all my problems with others, no, not that*” (SAH/13/D) “*‘*[nursing technician name]*, I’m upset’, he: ‘tell me about it.’ Then I tell it, I vent out. Then it seems to relieve a little, you know?*” (SAH/7/B)
Trust for clarification of doubts and exchange of information	“*Until then, I didn’t want to have children because I thought my child could be born with the virus and be sick and I chose not to. But after I was informed, the doctor told me that it was not like that, that I could have a child, that my child would come normal because I was a healthy person, my viral load has always been undetectable. Then I decided to have a baby, and I even delivered at the age of 40*” (PLH/4/A)	“*I only talk if they listen to me, because I think like this: if you are consulting the doctor, if you go to a medical facility, the professional should listen to you, to say the least. And, unfortunately, there are people who don’t listen to you, already take the pen, do it like this, that’s it, it’s over. They don’t even look at your face*” (SAH/21/E)
Knowledge coproduction, health literacy and self-care	“*If I search* [information about HIV on the internet]*, I double check with the doctor* [infectious disease specialist]*. She informs me of everything, you know? I’ve searched the internet already, but I can ask her everything. I don’t even need to search*” (PLH/19/C) “*At the time* [of the diagnosis]*, I went crazy, of course. You say: ‘wow, I won’t be able to have relations anymore.’* [...] *But gradually I got to know people who have it too.* [...]*. So I started to see the world from another perspective. If I took care of myself, always kept my viral load zero, I would take a minimal risk of contaminating other people*” (PLH/9/E)	“*I only see it* [the blood pressure] *when it hits the head, when the back of the head is throbbing or something, then I come here. It was supposed to be measured every three days, but I don’t come*” (SAH/8/B) “*It's the doctor and the device that I have, the one I use. They tell me how much pressure I have to have*” (SAH/18/C) “*Oh, I have a lot of friends* [laughs]*. Then they keep sending messages in WhatsApp. There are times when I’m like, 'Oh, I can’t take it anymore. They only think about illness, these people.’ ‘For cholesterol, take this, take that.’ But I don’t take anything...*” (SAH/34/F)
Availability of devices to receive information, appointment reminders, among others and access to contacts/telephone numbers of health care providers/services involved in care	“*After this professional retired* [nurse]*, I don’t have a landline, I don't have an email address, I don’t have a WhatsApp number, I do not have anything that I can talk to anyone in this sector*” (PLH/28/B)	“*Because that is all very loose: ‘it’s not ready, keep coming* [to the BHU to get information]*.’ These things, loose like that, discourage me.* [...] *We pay for it, you know? It discourages me, this very loose thing*” (SAH/30/H)

BHU: basic health unit; PLH: person living with HIV; SAH: users with systemic arterial hypertension.

Source: prepared by the authors based on the interviews.

Some SAH users felt comfortable talking about health problems with other FHT professionals, especially those with lower turnover. Regarding communication and familiarity, nursing technicians were noted as an important support for relationships and dialogs addressing issues beyond the health problem. When users perceived the absence of empathy in any type of health care service, they withdrew. In general, provider turnover and the COVID-19 pandemic affected the construction of dialogical relationships in PHC (Box 3). A recurring pattern in the accounts showed this perception: “*I don’t even know who is here* [BHU] *now*” (SAH/02/A).

The PLH mentioned the high number of appointments as one of the factors that made communication difficult, due to decreased available time. Few of them did not clearly understand examinations, adverse effects or recommended treatments and followed unrestrictedly the instructions of health care professionals, who guided important decisions in their lives. In addition, one participant, a travesti, considered the consultation time short to clarify doubts, for example, about adverse effects related to the use of antiretroviral therapy (ART) (Box 3).

Collective actions geared toward the production of health literacy and self-care in the health services were absent in the accounts of almost all participants. As SAH-related self-care practices, just over half reported monitoring blood pressure at home with the use of digital devices, without regularity. As for the PLH, they received instructions on eating and performing physical activities, at different frequencies between polyclinics. Several of them reported trying to adopt such instructions as a means of preventing diseases caused by the continuous use of antiretrovirals. Most of them had easy access to free prophylactics. There were common reports of association between the use of ART as self-care to ensure good health, long life, undetectable viral load and prevent sexual transmission (Box 3).

Although some of the users mentioned physicians, nursing technicians and CHWs as reference professionals for monitoring SAH, in different intensity, and infectious disease specialists and nurses in the case of PLH, this relationship was limited to clinical care provided in BHUs and polyclinics. There were few reports on guidance on SAH during CHW visits or through some collective or educational activity. In both cases - SAH and PLH -, individual approaches occurred during consultations and were highly valued by users (Box 3).

Three younger participants with SAH reported the internet as a source of health information. In disagreement, part of them reported difficulties in using it or lack of trust in the information. As for the PLH, half of them used the internet as an important source of health information on HIV/AIDS, with searches related to cure, vaccination, new therapies and scientific research, prejudice, food, among other topics. The others considered the infectious disease specialist, television, magazine, newspaper and friends - some, too, living with HIV - as reliable sources. Several of them reported consulting websites they considered reliable and the infectious disease doctor to avoid misinformation news. On the other hand, difficulties in accessing other health care professionals in the HCN caused procedures to be performed without adequate guidance based on information from the internet (Box 3).

No SAH or PLH participant mentioned institutional strategies or devices for receiving information. In the case of participants diagnosed with SAH, information on appointments or exams required a home visit of the CHW or, in many cases, consecutive visits to the BHU (Box 3). The participants did not identify formal communication channels (phone, message or email) for contact with a reference health care provider or service. This lack was a cause of complaint for some PLH, as they were not notified of canceled appointments or needed to reschedule them in person. In some polyclinics, there was such contact, conducted informally by nurses, through personal WhatsApp messages, with interruption when the professionals were replaced. The application was used as a communication channel by few infectious disease specialists for clinical guidance between consultations, on their own initiative.

## Discussion

The findings reinforce the importance of relational technologies as predictors of health outcomes, comparing diversified user experiences in PHC and specialized care in order to trace, at both health care levels, factors that facilitate and hinder the achievement of continuity of care. The results indicate aspects that differentiate and approximate the different experiences. In common, trust-based relationships and protagonism in the conduction of health care trajectories were established with the FHT physician or infectious disease specialist. Among the differences, according to SAH users monitored in PHC, relationship bonds were broken by health care provider turnover. In the absence of relationships with other FHT professionals, continuity and access were simultaneously affected. Among the PLH, the most significant characteristic was the strong trust-based relationship built with the infectious disease doctor, who had higher stability in the health care service. In the cases of the two conditions, the results showed a care with little concern for interprofessional practices and collective actions to strengthen continuity. Anyway, the study results indicate that relational continuity is important, and that it is highly valued by users.

The health-disease process has an unnatural history ^25^, whose sharing with the health care professional is part of the construction of trust-based relationships, which go beyond biological aspects ^26^. Accordingly, conditions permeated by stigma and prejudice, such as HIV, that determine inequalities of different orders - gender-related, social, among others ^27^ -, when shared with the professionals, can represent an important warning sign for changes in their practices, which often make invisible such dimensions of the health-disease-care process ^28^.

Trust is a psychological construct that influences health behaviors related to adherence to guidelines, prescribed medications and preventive measures ^29^, which are essential in the management of chronic conditions. Consultations with the same provider help develop a relationship of trust and reliability while unfamiliar settings and providers can generate anxiety and stress ^3^ ^,^ ^4^. Despite an a priori institutional trustworthiness granted to physicians, it can be limited and weakened by several factors ^3^, as observed in the experiences of SAH users, affected by the turnover of providers.

In addition, continuity enables health care providers to understand patient hopes, fears and desires, thus improving their clinical and personal skills ^3^, as seemingly indicated by the experience of PLH and not observed in the case of SAH users due to health care provider turnover. Consistently, when a health care provider learns about their patient, they increase their ability to perceive non-obvious or non-verbally expressed needs, to provide better care in emergencies, in addition to minimizing the repetition of the clinical and life history at each new meeting, an aspect that provides improved security and trust to users ^4^ ^,^ ^30^. The repetition of the clinical history at each new meeting, as found in the experience of people with SAH, is a cause of major dissatisfaction ^31^. Accordingly, to ensure care consistency and improve patient experience, it is necessary that clinical information is shared among health professionals in a timely manner ^32^, which can be delayed by the repetitive recording of the same information.

As mentioned, all the possibilities of strengthening the different dimensions of relational continuity lose power in the face of health care provider turnover, especially physician turnover, an issue not addressed in work management in SUS ^33^ and present in the most diverse contexts pari passu to the growth of PHC services ^4^. In addition to continuity, turnover compromises access to health care services, because, due to the expectation of non-provision of services, users no longer seek them ^34^, as found in the experience of people with SAH in this study.

Health care provider turnover made difficult the monitoring of users with SAH, even though care remained excessively centered on the physician. Other providers, such as nursing technicians and CHWs, had a secondary role, limited to mediating access, especially by scheduling appointments and exams and performing procedures. Thus, even when present in the health care setting, the other functions were subsumed to the biomedical logic ^35^.

No less relevant, in the case of PLH and SAH users, was the secondary role attributed to the work of nursing professionals in the sharing of clinical health care. Nurses in transitional care programs reduce re-hospitalizations, costs and improve the quality of life of people with chronic diseases ^36^. In addition, they work in the coordination and continuity of care, planning post-discharge care, coordinating points of care, conducting health education activities, managing complex cases, evaluating clinical and social aspects, and facilitating users’ navigation through health care services ^12^ ^,^ ^14^. From this perspective, promoting multiprofessional care both in PHC and specialized care, with protagonism of the role of nurses ^37^, seems to be a means to strengthen the coordination of care in the HCN and contribute to the users’ perception of continuity.

The bond with nursing professionals in hospitality was highly valued, especially among PLH and SAH users. As a soft care technology ^38^, hospitality favored adherence to treatment and the feeling of being cared for. The Cuban doctors’ approach was noted for the “kindness” and comprehensiveness, reinforcing the importance of these skills for the bond and continuity of care ^13^ ^,^ ^39^ and that, therefore, they cannot be considered secondary.

Health literacy is a multidimensional process that encompasses individual and collective skills to seek, evaluate and interpret health-related information, for self-care or the care of others ^40^. In the study with PLH, infectious disease specialists centralized the management of information crucial to the health of users. People with SAH also valued the centrality of physicians, even though the bonds were weakened. In both groups, individual consultations were the main space for health guidance, although this space is recognized by the asymmetry of power and in which the expectation for the resolution of health problems is directed to health care providers ^10^. Collective health education practices ^40^, or even external ones, during the visit of CHWs, could be strategic to expand literacy from a perspective that would include diverse experiences, with sharing of knowledge and care practices.

The availability of personalized information is essential for relational continuity and maintains a direct relation with the informational domain. However, the absence of information and communication technologies (ICT), as found in this study, makes it difficult to provide person-centered care. ICTs have potential to improve the quality, sharing and accessibility of information for users, such as by minimizing the need for in-person visits ^41^.

Relational continuity strengthens security and avoids the repetition of clinical histories, facilitating navigation in the health care system ^30^. This study found no formal strategies, such as STP, to enable the contact and sharing of therapeutic plans between users and professionals of the HCN, with predominance of informal initiatives. These aspects concomitantly indicate weaknesses in the domains of managerial and informational continuity. In these cases, although patients and family members are assigned the position of single link between different points of care with the mission of facilitating continuity of care, this responsibility does not come with the necessary skills or confidence ^42^.

The results of this study confirm that continuity cannot be analyzed while assuming that other attributes of health care systems and services have been fully achieved. A broad understanding that can be translated into policies, actions and practices to achieve continuity requires a consideration of the global context, especially with regard to access to and quality of health care ^21^.

As limits of the study, it is noted that the complexity inherent in the care of people with complex needs requires mechanisms at the individual level that ensure relational continuity, as well as actions within the organizational scope of the health care system that are capable of mitigating health care fragmentation and that were not the focus of this study. Ensuring informational and managerial continuity is part of this broad set of strategies, which are not directly addressed in this article, albeit interrelated. In any case, it is argued that the diversity of experiences, based on the context of the participants in different sets of care, provide a dense analysis of the situation, which does not exclude other experiences and interpretations.

## Final considerations

At the interface between the population and the health care system, health care providers must conduct their practice centered on the person in order to enable a relationship of therapeutic trust and reciprocity. The results of this article indicate the importance of a regular point of care for achievement of the bond and consequent relational continuity. On the other hand, health care work management-related issues contribute to health care provider turnover and sequential disruptions in therapeutic follow-up with detrimental impacts on all - users, professionals and managers. It is also argued that continuity of care needs to be transversal to the HCN and is strengthened as multiprofessional teams are involved in the production of coherent, coordinated therapeutic projects in synergy with the needs and interests of users. In the case of chronic and long-term diseases, the loss of relational continuity compromises adherence and loyalty, leading to reaggravations, abandonment of treatment, withdrawal from seeking care or seeking private and emergency care services, thereby increasing inequalities among more vulnerable groups.
