# Supplementary material for: Continuity of care: trust-based relationship and availability of personalized information in user experience
Source: Cad Saude Publica. 2025 Jul 18;41(6):e00109524. doi: 10.1590/0102-311XEN109524 (PMC12315564; doi:10.1590/0102-311XEN109524)
Supplement: Translation

# Translation: Continuidade do cuidado: relações de confiança e disponibilidade de informações personalizadas na experiência de usuários

**DOI:** 10.1590/0102-311XPT109524

**Authors:** Patty Fidelis de Almeida, Adriano Maia dos Santos, Rafaela Fidelis Lima Silvério, Amanda Maria Villas Bôas Ribeiro, Drieli Oliveira Silva, Ana Luiza Queiroz Vilasbôas

**Affiliations:** 1 Instituto de Saúde Coletiva, Universidade Federal Fluminense, Niterói, Brasil.; 2 Instituto Multidisciplinar em Saúde, Universidade Federal da Bahia, Vitória da Conquista, Brasil.; 3 Instituto de Saúde Coletiva, Universidade Federal da Bahia, Salvador, Brasil.

**Keywords:** Continuidade da Assistência ao Paciente, Integralidade em Saúde, Acessibilidade aos Serviços de Saúde

## Abstract

Este artigo analisa a continuidade do cuidado no domínio relacional a partir da experiência de usuários. Foi realizado um estudo de caso qualitativo com base em 45 entrevistas com pessoas que vivem com HIV (PVH) acompanhadas em policlínicas e 38 com usuários com diagnóstico de hipertensão arterial sistêmica (HAS) cadastrados em unidades básicas de saúde (UBS) em um município de grande porte do Estado do Rio de Janeiro, Brasil. Os resultados são analisados a partir de duas dimensões da continuidade relacional: as relações de confiança e a disponibilidade de informações personalizadas. Junto ao médico da equipe de saúde da família (EqSF) e ao infectologista nas policlínicas se estabeleciam as mais fortes relações de confiança e o protagonismo na disponibilização das informações em saúde. Entre as diferenças, para os usuários com HAS, os vínculos foram cindidos pela rotatividade médica. Na vacância de relações com outros profissionais das EqSF, a continuidade e o acesso foram simultaneamente afetados. Entre as PVH, profissionais de enfermagem desempenhavam importante papel no acolhimento. Nos dois casos, os resultados revelaram um cuidado pouco afeito às práticas interprofissionais, ações coletivas e promocionais no fortalecimento da continuidade. A ausência de comunicação intermediada por tecnologias exigia dos usuários idas aos serviços para resolução de suas demandas e contato com os profissionais. Os resultados sinalizam a importância de um ponto regular de cuidado para a consecução do vínculo e a consequente continuidade relacional. Por outro lado, problemas relacionados à gestão do trabalho no Sistema Único de Saúde intensificam a rotatividade profissional e favorecem as rupturas no seguimento terapêutico.

## Introdução

A continuidade do cuidado tem sido associada à redução das internações, da mortalidade e do uso de emergências, além de fortalecer a adesão à promoção da saúde e a relação entre profissionais e usuários ^1^ ^,^ ^2^ ^,^ ^3^ ^,^ ^4^. Seu alcance representa um grande desafio aos sistemas de saúde em todo o mundo, cuja implementação de políticas e ações concentra-se no âmbito da atenção primária à saúde (APS) ^4^ ^,^ ^5^. Contudo, para as pessoas que convivem com agravos crônicos ou com necessidades em saúde complexas, a garantia de continuidade envolve serviços especializados, hospitalares, sociais e comunitários ^6^ ^,^ ^7^.

A clássica concepção de Haggerty et al. ^8^, amplamente adotada e adaptada a diversos contextos, propõe uma estrutura de continuidade do cuidado em três domínios: informacional, gerencial e relacional. O primeiro pressupõe o uso contínuo de informações relevantes sobre o histórico do paciente e suas condições de vida e saúde com vistas a tornar o cuidado atual e futuro adequados. A continuidade gerencial envolve a coerência no manejo clínico a partir da cooperação e comunicação entre os diversos prestadores para oferta de cuidados em tempo hábil e sem duplicidades. O domínio da continuidade relacional, foco deste artigo, envolve a qualidade e a coerência da relação produzida entre profissionais/equipe de saúde e usuários ^8^ ^,^ ^9^.

É inegável a interdependência entre os três domínios. Argumenta-se que, embora a continuidade seja um constructo multidimensional, precisa ser considerada no processo de organização dos serviços de saúde e, sobretudo, na experiência dos usuários ^10^. Para usuários, familiares e cuidadores a continuidade é percebida e facilitada quando recebem apoio dos profissionais ou equipes que passam a assumir a responsabilidade pelos planos terapêuticos e oferecem suporte no manejo dos diversos recursos (medicamentos, tratamentos, funcionamento do sistema de saúde) ^11^.

A continuidade relacional posiciona-se na dimensão micro do sistema de saúde. Implica o estabelecimento de relacionamentos duradouros e personalizados entre profissionais/equipes e usuários, além de uma abordagem holística dos problemas de saúde ^4^. Representa mais do que “ter contato com o mesmo médico”, mas um compromisso bidirecional baseado na confiança mútua e na responsabilidade do profissional de saúde com seus pacientes, cuja intensidade aumenta à medida em que as relações se tornam mais estáveis e sustentáveis ^10^. Nesta perspectiva, a continuidade se aproxima do conceito de “longitudinalidade” ao incorporar o estabelecimento de relações de confiança ao longo do tempo com um prestador reconhecido como fonte regular de cuidados ^12^.

Sidaway-Lee et al. ^3^ destacam que a continuidade relacional apresenta efeitos sobre os resultados em saúde por meio de mecanismos como o contato frequente, o conhecimento acumulado, o senso de responsabilidade do profissional, a qualidade da relação, a confiança, a empatia. A estabilidade da equipe de saúde favorece essa continuidade, permitindo relações baseadas nas necessidades dos usuários (clínicas, mentais, sociais e ambientais) e no acesso adequado aos recursos disponíveis ^13^. Nesse sentido, favorece a adesão e a confiança nas orientações dos profissionais ^11^, que por sua vez, com o contato prolongado, desenvolvem uma visão mais abrangente do cuidado ofertado nos diversos serviços de saúde e do papel dos familiares ^4^.

No Brasil, a desigualdade social e as dificuldades na operacionalização das Redes de Atenção à Saúde (RAS) apresentam efeitos sobre a continuidade do cuidado que, frequentemente, depende de mecanismos informais empreendidos por usuários e familiares ^14^. Estudos brasileiros ^12^ ^,^ ^15^ ^,^ ^16^ apontam fragilidades na relação interpessoal entre usuários e profissionais, possivelmente relacionadas a dificuldades de acesso à APS. Ainda assim, um conjunto de estratégias e ferramentas para a continuidade do cuidado foram identificadas, como: alta segura, gestão da clínica, discussão de casos, linhas de cuidado, enfermeira de enlace, complexos reguladores e educação permanente (ainda que não necessariamente implementados no país) ^17^. Todavia, permanecem pouco explorados arranjos organizativos que catalisem processos de trabalho numa perspectiva interprofissional para efetiva continuidade relacional e fluxo comunicacional compartilhado, especialmente em cenários de vazios assistenciais ^18^, territórios conflagrados ^19^ e para populações cuja abordagem em saúde requer maior competência cultural ^20^.

Este artigo analisa a continuidade do cuidado no domínio relacional a partir da experiência de pessoas que vivem com HIV (PVH) cujos cuidados são centralizados em serviço especializado e usuários com hipertensão arterial sistêmica (HAS) cadastrados em unidades básicas de saúde (UBS). Busca-se responder à seguinte pergunta de investigação: considerando diferentes contextos de atenção à saúde (serviços especializados e UBS), quais fatores favorecem ou dificultam a continuidade relacional na perspectiva dos usuários? Ambos os agravos, pelo caráter de cronicidade, exigem acompanhamento contínuo que contribua na adesão aos cuidados individuais e coletivos. Argumenta-se que, a despeito dos avanços técnicos, principalmente das tecnologias biomédicas, fatores relacionais continuam sendo importantes preditores de resultados em saúde ainda que, contraditoriamente, haja uma diminuição do valor atribuído ao contato pessoal entre equipes, profissionais e usuários ^1^. Além disso, cotejar experiências diversificadas em diferentes tipos de prestadores e agravos em saúde aprofunda a compreensão sobre a continuidade do cuidado, sendo esta uma das principais contribuições deste estudo. Na mesma direção, a maioria dos conceitos e modelos de análise baseia-se em estudos de países de alta renda, contrastando com a realidade de contextos com recursos limitados e alta fragmentação, como o brasileiro ^14^ ^,^ ^21^.

## Metodologia

### Tipo de estudo

Foi realizado o estudo de caso, com abordagem qualitativa, com base em entrevistas de usuários diagnosticados com HAS, cadastrados por equipes de saúde da família (EqSF), e PVH acompanhadas em policlínicas. Foram reconstruídas as trajetórias assistenciais dos participantes na busca por atenção à saúde da APS aos demais pontos da RAS, inclusive no setor privado. Para este artigo, foram identificados e analisados resultados que informavam sobre o domínio relacional da continuidade do cuidado.

### Cenário do estudo e critérios de seleção dos participantes

O cenário do estudo foi um município localizado no Estado do Rio de Janeiro, Brasil, com população aproximada de 500 mil habitantes.

Foram realizadas 38 entrevistas presenciais com usuários com HAS, de fevereiro a julho de 2022, em 10 das 43 UBS; e 45 entrevistas com PVH, entre dezembro de 2021 a junho de 2022, nas sete policlínicas municipais de especialidades que, no cenário do estudo, são os principais serviços de acompanhamento.

A seleção priorizou UBS com 3 a 6 EqSF, distribuídas nas sete regionais de saúde, devido à pandemia de COVID-19, que reduziu o fluxo de usuários, dificultando a probabilidade de encontrá-los em serviços com menor número de equipes. Além disso, a seleção das UBS com este perfil considerou a vacância/troca de profissionais, resultante de concurso público no período do estudo.

Após a seleção das UBS, possíveis participantes foram identificados pelos entrevistadores com auxílio das respectivas EqSF, conforme os critérios de inclusão: ter diagnóstico de HAS e estar cadastrado por uma EqSF; solicitação de referência pela APS para consulta ou exame especializado nos últimos 12 meses anteriores à entrevista (verificado nos prontuários ou livros de registro); ter 18 anos completos ou mais; e não apresentar comprometimento físico ou psicológico impeditivos à realização da entrevista (segundo avaliação dos profissionais das EqSF). Desta seleção inicial, que variou em cada uma das UBS selecionadas, os potenciais participantes foram convidados a comparecer ao serviço ou consultados sobre a possibilidade da realização da entrevista em domicílio.

Em relação às PVH, foram entrevistados usuários vinculados às sete policlínicas municipais, com base nos critérios de inclusão: ter 18 anos completos ou mais; residentes no município; com testagem positiva para HIV há pelo menos 1 ano anterior à realização da entrevista. Os participantes foram identificados com o auxílio de profissionais do serviço e convidados a participar durante a espera ou logo após a consulta com o infectologista.

### Produção de dados

As entrevistas foram realizadas por pesquisadores previamente capacitados e guiadas por um roteiro semiestruturado. Em relação aos dois agravos, o roteiro de entrevista continha os seguintes blocos: perfil sociodemográfico, hábitos e estilo de vida, descoberta do diagnóstico, cuidado recebido na APS e na atenção especializada (AE), outros caminhos percorridos no Sistema Único de Saúde (SUS) e na rede privada, cuidados durante a pandemia de COVID-19 e avaliação geral da atenção recebida. A continuidade do cuidado, tal como compreendida neste artigo, é o “amálgama” que favorece a percepção pelos usuários do *continnum* assistencial e foi abordada ao interior das experiências de cuidado em cada nível ou serviço de saúde e na transição entre eles. As entrevistas abordando a HAS, com média de 30 minutos, e as entrevistas com PVH, com média de 1 hora, foram gravadas, transcritas na íntegra para análise e interpretação dos resultados. A finalização da produção dos dados foi determinada a partir do alcance de informações adequadas e suficientes para a compreensão do fenômeno ^22^, compostas por relatos de usuários das sete regionais de saúde do município.

### Análise dos dados

Para análise dos resultados, realizou-se a ordenação geral dos dados. As transcrições foram lidas exaustivamente e os núcleos temáticos foram categorizados a partir das dimensões pré-estabelecidas para continuidade relacional. A interpretação foi orientada pela análise de conteúdo temático, identificando-se convergências e divergências ^23^. Ademais, os excertos mais representativos foram selecionados e distribuídos nas respectivas categorias.

Para descrição e interpretação dos resultados, partindo do referencial de Haggerty et al. ^8^ e referências complementares ^3^ ^,^ ^4^ ^,^ ^10^ ^,^ ^11^ ^,^ ^21^ ^,^ ^24^ foram identificadas, previamente, dimensões vinculadas ao domínio da continuidade relacional - relações de confiança e disponibilidade de informações personalizadas - e suas respectivas definições, apresentadas no Quadro 1. Na sequência, os relatos produzidos a partir das entrevistas, foram cotejados às dimensões para definição e adequação das ações/atividades que compõem a matriz de análise (Quadro 1).

**Quadro 1:** Dimensões, definição e ações/atividades da continuidade relacional na experiência dos usuários.

DIMENSÕES	DEFINIÇÃO	AÇÕES/ATIVIDADES PERCEBIDAS PELOS USUÁRIOS
Relações de confiança entre profissionais/serviços de saúde e usuários	Pressupõe o estabelecimento de relações terapêuticas estáveis e personalizadas entre profissionais/equipes e usuários e uma abordagem holística dos problemas de saúde que considere seus recursos, preferências e necessidades. Envolve a responsabilidade da equipe/profissional com os usuários. Nessa perspectiva, a confiança influencia o comportamento dos usuários na melhor adesão às orientações, aos medicamentos e medidas preventivas	Identificação de um profissional de referência/coordenador do cuidado Percepção de que o profissional/equipe de saúde tem uma visão holística de sua história clínica/pessoal Confiança no profissional/equipe de saúde Projeto terapêutico singular que oriente a conduta dos profissionais da equipe de atenção primária à saúde e demais envolvidos
Disponibilidade de informações personalizadas	Refere-se à comunicação com troca de informações claras sobre a condição de saúde, adaptadas às capacidades e necessidades únicas de cada usuário, respeitando-se, inclusive, os tempos individuais para lidar com as situações de adoecimento/tratamento. Inclui considerar o conhecimento prévio ou compreensão que cada usuário tem sobre seu agravo	Recebimento de orientações claras, compreensíveis e personalizadas Confiança para o esclarecimento de dúvidas e troca de informações Coprodução de conhecimento no desenvolvimento de literacia em saúde e autocuidado Disponibilidade de dispositivos para recebimento de informações, lembretes de agendamentos, entre outros Acesso aos contatos/números de telefone de profissionais/serviços de saúde envolvidos no cuidado

Fonte: construído com base em Haggerty et al. ^8^; Ljungholm et al. ^4^ ^,^ ^11^; Nóbrega et al. ^24^; Hill et al. ^10^; Meiqari et al. ^21^; Sidaway-Lee et al. ^3^.

O modelo proposto, embora busque identificar elementos afeitos ao domínio da continuidade relacional deve ser considerado à luz da relação entre os demais componentes - informacional e gerencial, uma vez que são dependentes e interelacionados ^18^.

O estudo foi aprovado pelo Comitê de Ética em Pesquisa do Instituto de Ciências Humanas, Universidade Federal Fluminense (parecer nº 4.456.756), com anuência do município. Os participantes foram identificados por sigla (PVH ou HAS), número que corresponde à realização da entrevista e uma letra atribuída a cada serviço - policlínica no caso HIV e UBS no caso HAS.

## Resultados

### Perfil dos entrevistados

A Tabela 1 apresenta o perfil dos participantes do estudo. Entre as PVH, cerca da metade eram homens e a faixa etária concentrou-se entre 41 a 60 anos, com parceiros e filhos. A grande maioria se identificava como preto e pardo (75,5%), com Ensino Médio (44,4%), renda familiar de até 2 salários mínimos (53,3%) e provedores do lar (53,3%). Já entre os 38 participantes com diagnóstico de HAS, predominaram indivíduos do gênero feminino (76,3%), autodeclarados preto e pardo (71%) e com idade acima de 60 anos (55,3%). Em sua maioria, eram os principais provedores do lar (60,5%), com renda familiar de até 1 salário mínimo (52,6%). Metade dos participantes com HAS (50%) recebia algum tipo de benefício social ou previdenciário. Em ambos os grupos, a maioria não possuía planos de saúde, sobretudo entre usuários com HAS, que também apresentavam menor renda (Tabela 1).

**Tabela 1:** Caracterização dos participantes do estudo. Município de grande porte, Rio de Janeiro, Brasil, 2022.

Características socioeconômicas	PVH		HAS	
	n	%	n	%
Idade (anos)				
< 49	29	64,5	6	15,7
50-59	10	22,2	11	28,4
60-69	4	8,9	13	34,2
70-79	2	4,4	7	18,4
80 ou mais	0	0,0	1	2,6
Raça/Cor (autodeclarada)				
Branca	8	17,8	11	28,4
Parda	22	48,9	18	47,3
Preta	12	26,7	9	23,6
Indígena	0	0,0	0	-
Amarela	2	4,4	0	-
Prefiro não responder	1	2,2	-	
Gênero				
Homem cis	23	51,1	8	21,1
Mulher cis	18	40,0	29	76,3
Travesti	1	2,2	1	2,6
Não binário	2	4,4	-	-
Prefiro não responder	1	2,2	-	-
Escolaridade				
Fundamental incompleto	3	6,7	11	28,4
Fundamental completo	3	6,7	11	28,4
Médio incompleto	7	15,6	5	13,2
Médio completo	20	44,4	10	26,3
Superior incompleto	3	6,7	1	2,6
Superior completo	9	20,0	-	-
Renda familiar (salários mínimos)				
< 1	-	-	20	52,6
1-2	23	51,1	10	26,3
Até 3	11	24,4	5	13,2
Até 4	7	15,6	2	5,3
Mais que 4	2	4,4	-	-
Refere não ter renda	1	2,2	1	2,6
Não sabia	1	2,2	-	-
Benefício social *				
Não	30	66,7	19	50,0
Sim	15	33,3	19	50,0
Aposentadoria/Pensão	5	-	7	36,8
Benefício de Prestação Continuada (BPC)	0	-	1	5,3
Benefício Social Municipal	3	-	7	36,8
Auxílio Brasil/Emergencial	7	-	4	21,1
Bolsa Família	6	-	-	-
Plano de saúde				
Não possui	28	62,2	31	81,6
Plano de saúde	6	13,3	2	5,3
Plano popular	11	24,5	5	13,1

HAS: usuários com hipertensão arterial sistêmica; PVH: pessoas que vivem com HIV.

Fonte: elaboração própria baseada nas entrevistas.

* Algumas PVH relataram receber mais de um benefício, portanto, não foi possível calcular o percentual dos benefícios específicos.

### Relações de confiança entre profissionais/serviços de saúde e usuários

O médico da EqSF era a principal referência para o acompanhamento dos usuários com HAS ao longo do tempo, embora em função da seleção pública ocorrida à época e pelo contexto de pós-pandemia de COVID-19, muitos profissionais já não faziam parte das equipes. Os usuários se ressentiam pelas mudanças e explicitavam que “*aqui* [UBS] *não tem continuidade para nada*” (HAS/30/H). Um conjunto expressivo de participantes reforçou os efeitos deletérios da rotatividade profissional na ruptura de vínculos e na desistência de busca pelo serviço de saúde (Quadro 2). No mesmo contexto das queixas sobre a rotatividade médica, narrativas espontâneas sobre a relação estabelecida com médicas cubanas do Programa Mais Médicos que atuaram em algumas UBS, até 2018, foram recordadas. Aspectos definidos como “delicadeza”, atenção e visão integral do sujeito foram relacionados à satisfação e à sensação de estar sendo cuidado (Quadro 2).

**Quadro 2:** Relações de confiança entre profissionais/serviços de saúde e usuários - falas expressivas dos participantes. Município de grande porte, Rio de Janeiro, Brasil, 2022.

AÇÕES/ATIVIDADES	FALAS EXPRESSIVAS	
	PVH	HAS
Identificação de um profissional de referência/coordenador do cuidado	“*Qualquer coisa que eu sinto, é com ela* [infectologista] *que eu falo* [...] *A minha vida* [risos]*. Coordenam a minha vida...*” (PVH/16/D) “*...Sofri muito. Porque dos meus 9 anos até os meus 16 ou 17 anos, eram sempre as mesmas pessoas, e quando eu vi ir embora, foi ali que acho que começou a desandar tudo da minha vida, em relação a tratamento. Porque eu era muito apegada à psicóloga, à própria médica, à assistente social, às enfermeiras. Aí, quando eles foram embora, eu sofri. Não era a mesma coisa. Eu ia para a consulta desarrumada, tipo, ia assim por ir. Porque antes, quando eles estavam, eu fazia questão de ir toda arrumada para vê-los*” (PVH/5/A) “[A ACS] *é minha vizinha, mas não tenho contato com ela, não. Quando precisei, evitei, porque, também, não queria a rua inteira sabendo que eu estava com HIV, ainda mais depois de ter me separado. Seria horrível para mim*” (PVH/45/A)	“*Agora eu nem tenho ido. Não tem médico no posto... Ficava uns dois, três meses, depois saía. Ficava uns dois meses sem médico, três meses. Que nem tá agora, não tem médico. Só atende a gente quando a gente chega lá muito ruim*” (HAS/20/C) “*Aí teve muita troca de médico, teve uma época que... assim, estava... aí eu comecei a verificar em casa mesmo. Eu falei assim: ‘oh, se estiver acima de 14, eu vou para o médico*’” (HAS/36/J) “*É, aquela médica cubana. Ai, adorava ela! Ela era muito boa, sabe? Atendia você, a gente não entendia muito a voz dela, mas atendia a gente numa delicadeza total.* [...] *E ela de três em três meses me via...*” (HAS/31/H) “*Ela era boa porque: ‘oh, não vou tratar a sua doença, vou tratar de você’. Amei essa frase dela... Então, ela me examinou de cabo a rabo*” (HAS/30/H) “*‘*[nome]*, eu não tô podendo...’, aí ele* [ACS] *marcava com a doutora* [nome] *e eu vinha. ‘Ah, vem que eu vou marcar’. Ele era ótimo. Agora ele não tá mais aqui...*” (HAS/4/A) “*Eu falo: tá acontecendo isso, assim, assim ‘*[técnica de enfermagem] *eu vou dar um jeitinho!’ Então é uma pessoa que a gente tem mais acesso, entendeu? Precisou, corre lá no* [nome técnica de enfermagem]” (HAS/13/D)
Percepção de que o profissional/equipe de saúde tem uma visão holística de sua história clínica/pessoal	“*Eu acho que ela* [infectologista] *é a pessoa que mais sabe de mim. Igual Big Brother, as pessoas vão para o Big Brother para se conhecer. E ela é a pessoa que mais sabe de mim*” (PVH/37/C) “*Os que eu mais confio são tanto a doutora* [nome do infectologista] *quanto esse enfermeiro que veio aqui, porque ele também tá sempre acompanhando de perto, pergunta se tá tudo bem, se precisa de alguma ajuda, como é que tá o remédio, se eu preciso repor*” (PVH/15/D) “*É, eu tenho muito carinho por elas* [equipe de enfermagem]*. Na verdade, é mais gratidão pelo que elas fazem. Elas ajudam bastante a gente. Assim, a gente não tem só o apoio médico, sabe? É um apoio emocional que a gente também precisa. Aqui é bom por isso*” (PVH/1/B)	“*Agora tá tudo novo, mudou tudo, eu não tenho intimidade, não conheço ninguém porque eu quase não venho*” (HAS/8/B) “*Mas é aquilo, são pessoas diferentes que não tem aquele cotidiano da vida da gente. Os médicos anteriores são médicos antigos que conheciam a gente. Hoje em dia, os médicos não conhecem os pacientes daqui. Então fica bem complicado passar certas questões*” (HAS/2/A)
Confiança no profissional/equipe de saúde	“*Eu tenho concentrado meu cuidado muito nela* [infectologista]*. Eu deixei minha vida nas mãos dela, então ela sempre sabe o que eu preciso, o que eu não preciso, ela sempre vê tudo direitinho*” (PVH/15/F) “*Tudo, exame de sangue, medicamento, consulta, outros tipos de doença, febre, tudo com ela. Ela é a doutora que eu entrego tudo na mão dela*” (PVH/36/C)	“*No momento, não venho aqui não* [UBS]*. Mudou tudo...* [...] *sabe que eu não sei te explicar isso?* [risos]*. Não sei se era mais atenção, tinha mais atenção.* [...] *Tipo assim, mais confiança, aí vinha mais: ‘não pode deixar de vir’, aí* [a médica] *aconselhava: ‘Para de fumar, diminui o cigarro...*’” (HAS/8/B)
Projeto terapêutico singular que oriente a conduta dos profissionais da equipe de atenção primária à saúde e demais envolvidos	Pergunta: E o médico de lá [APS], ele faz algum encaminhamento, algum laudo para você entregar para a médica aqui [policlínica]? “*Não*” Pergunta: E a doutora [infectologista] faz algum para entregar lá? “*Não. Aqui é aqui e lá é lá*” Pergunta: Os médicos se conversam? “*Não*” (PVH/27/B) “*Isso daqui já poderia ter melhorado nesse sentido. O médico queria saber todo meu histórico de HIV para depois saber como é que ele ia me tratar. E eu lá morrendo de dor* [em um serviço de pronto-atendimento]” (PVH/42/E)	“*Se eu não tiver uma linha de tratamento, eu vou ter uma emergência péssima. Que é o que estava acontecendo. Eu estava sempre indo para o hospital com pressão alta e nunca conseguindo fazer o mapeamento aqui* [UBS]” (HAS/11/C) “*Eu acho que se tivesse um intercâmbio, uma internet boa, um trabalho bom, quando eu chegasse com meu número de prontuário naquela especialidade, já tinha que tá tudo prescrito ali dentro. Você tem que repetir tudo de novo! São coisas que... eu não sou inteligente o suficiente, não fiz uma faculdade, mas eu sei o que incomoda. Entendeu?*” (HAS/11/C)

ACS: agente comunitário de saúde; APS: atenção primária à saúde; HAS: usuários com hipertensão arterial sistêmica; PVH: pessoas que vivem com HIV; UBS: unidade básica de saúde.

Fonte: elaboração própria baseada nas entrevistas.

De forma inequívoca, as PVH indicaram o infectologista como a principal referência para acompanhamento da saúde em geral, em alguns casos, a única. Destacou-se a intensidade do vínculo com o infectologista, reconhecido como o profissional para o qual se “*entregou a vida*” (PVH/15/F). Foram feitas menções ao acolhimento realizado pelos enfermeiros em quatro dos sete serviços. A regularidade no pré agendamento das consultas com o infectologista - trimestral ou semestral - foi apontada pelas PVH como favorecedora do vínculo e do acesso. A maioria dos entrevistados se considerou assíduo às consultas e reconhecia que, principalmente, infectologistas e enfermeiros tinham uma compreensão ampliada de suas histórias clínicas e de vida. Embora, à época, também tenha ocorrido seleção pública para profissionais das policlínicas, a rotatividade não apareceu de forma importante nos relatos, embora alguns usuários apresentassem receio em relação a possíveis trocas de profissionais com base em experiências pregressas (Quadro 2).

Os agentes comunitários de saúde (ACS) foram mencionados, por usuários com HAS, como uma referência importante pela intermediação das demandas apresentadas à UBS, comunicando sobre a possibilidade de marcação de consultas, situação dos agendamentos para os serviços especializados, chegada de exames, ausência de profissionais e atualização do cadastro (Quadro 2). No caso das PVH, não houve relatos sobre vinculação com os ACS. Pouco mais da metade dos participantes (26 PVH) referiu possuir cadastro em UBS e, apenas, quatro relataram utilizá-las regularmente. Quando ocorriam visitas domiciliares, comumente estavam direcionadas a familiares com problemas de saúde e, raramente, às PVH. As atividades relatadas foram similares a dos usuários com HAS, porém direcionadas a outros membros da família. Ademais, as PVH apontaram o receio sobre a quebra do sigilo diagnóstico como um empecilho para vinculação aos ACS (Quadro 2).

Na sequência, muitos participantes com HAS citaram os técnicos de enfermagem como referência, especialmente pela realização de procedimentos como aferição da pressão arterial, antropometria, glicemia capilar e acolhimento nas UBS. O enfermeiro foi referido em poucos casos, não sendo relatadas consultas clínicas para manejo da HAS. Entre as PVH, o profissional de enfermagem, em algumas policlínicas, tinha um papel destacado e valorizado pelos participantes no acolhimento e na realização de busca ativa (telefônica/mensagem) nos casos de absenteísmo. Estes profissionais organizavam o fluxo de atendimento interno no serviço, uma vez que em algumas destas unidades não havia consultórios fixos para as consultas e os usuários se sentiam perdidos e expostos (Quadro 2).

Os participantes com HAS enfatizaram que as histórias clínicas se perdiam em decorrência da rotatividade do médico, o que também rompia a experiência da “intimidade” (Quadro 2). Além da ruptura dos vínculos, os períodos de vacância agravavam problemas de acesso à APS. O acompanhamento da HAS ficava restrito, sendo comum as tentativas de “encaixes”, via demanda espontânea. Muitos usuários com HAS desistiam de buscar a UBS ou procuravam outras formas de cuidado como a aferição da pressão arterial no domicílio ou busca de consultas e exames em planos populares (Quadro 2).

No caso das PVH, a rotatividade não apareceu de maneira expressiva. Os participantes se consideravam vinculados às policlínicas e buscavam os profissionais para quase todos os problemas de saúde. Foram muito frequentes os relatos de busca do infectologista em situações como gripe, controle de hipertensão, entre outras necessidades. Muitos usuários apresentavam ao infectologista receitas e resultados de exames prescritos por outros profissionais - da APS, hospitais, unidades de pronto atendimento ou especialistas da rede privada - para fins de acompanhamento ou mesmo para revisão da conduta (Quadro 2).

Não foram relatadas pelos participantes com HAS e PVH as estratégias ou as ações que pudessem configurar a construção de um projeto terapêutico singular (PTS) entre a EqSF e as policlínicas, com a participação de outros profissionais ou serviços de saúde. Nas policlínicas, embora houvesse a possibilidade de referências internas a outros especialistas, não houve relatos de produção de PTS. A delegação da função de comunicação dos respectivos históricos clínico e de vida ficava sob responsabilidade dos usuários ou familiares, o que exigia lembrança, compreensão de todas as orientações e prescrições para transmiti-las nos diversos atendimentos, o que gerava insatisfação (Quadro 2).

### Disponibilidade de informações personalizadas

A principal fonte de informação para os cuidados com a HAS foi o médico. Alguns relatos sinalizaram que somente com este profissional era estabelecida a confiança necessária para esclarecer dúvidas ou trocar informações sobre os problemas de saúde. Para PVH, os infectologistas e os enfermeiros ocupavam esse lugar (Quadro 3).

**Quadro 3:** Informações personalizadas - falas expressivas dos participantes. Município de grande porte, Rio de Janeiro, Brasil, 2022.

CATEGORIAS	FALAS EXPRESSIVAS	
	PVH	HAS
Recebimento de orientações claras, compreensíveis e personalizadas	“*E ela* [infectologista] *me explica o porquê de tudo: ‘é por isso que estou retirando essa medicação e colocando essa medicação...’*” (PVH/18/C) “*É uma pena. Eu não consigo porque na saúde pública é muito difícil, é muita gente, uma fila gigantesca de gente para atender. A médica, se ela ficar 40 minutos comigo, conversando comigo, é demais. É muita gente do lado de fora esperando, estressada*” (PVH/17/C)	“*Com os médicos sim, com certeza. Os outros profissionais, assim, nem tanto... Elas sabem* [outras profissionais] *que eu tenho diabetes, que eu tenho pressão alta, mas que eu vou expor todo o meu problema com os outros não, isso aí não*” (HAS/13/D) “*‘*[nome técnico de enfermagem]*, eu tô chateada’, ele: ‘fala para mim’. Aí eu conto, desabafo. Aí parece até que alivia um pouco, entendeu?*” (HAS/7/B)
Confiança para o esclarecimento de dúvidas e troca de informações	“*Até então, eu não queria ter filhos porque eu achava que meu filho poderia vir com o vírus e ser doente e eu optei por não ter. Mas depois que eu fui informada, a médica conversou comigo que não tinha nada a ver, que eu poderia ter um filho que meu filho viria normal porque eu era uma pessoa saudável, minha carga viral sempre foi indetectável. Então eu resolvi ter um neném, tanto é que ganhei aos quarenta anos*” (PVH/4/A)	“*Eu só falo se me der atenção, porque penso assim: se você tá passando, se você vai num lugar, a pessoa tem que pelo menos te escutar. E, infelizmente, tem gente que não te escuta, já pega a caneta, faz assim, pronto, acabou. Nem olha no teu rosto*” (HAS/21/E)
Coprodução de conhecimento, literacia em saúde e autocuidado	“*Se eu buscar* [informações sobre HIV na internet]*, eu pergunto para doutora* [infectologista]*. Ela me informa tudo, entendeu? Eu já busquei na internet já, mas tudo eu posso perguntar a ela. Não preciso nem de buscar*” (PVH/19/C) “*Na época* [do diagnóstico]*, pirou a minha cabeça, claro. Você tira: ‘poxa, não vou poder mais me relacionar’.* [...]*, mas aos poucos fui conhecendo pessoas que têm também.* [...]*. Então eu passei a enxergar o mundo por outro lado. Se eu me cuidasse, sempre mantivesse a minha carga viral zerada, assumiria um risco mínimo de contaminar outras pessoas*” (PVH/9/E)	“*Eu só vejo* [a pressão arterial] *quando dá na cabeça, que a nuca tá dando algum sinal, aí eu venho. Que era para medir de três em três dias, mas eu não venho*” (HAS/8/B) “*É o médico e o aparelho que eu tenho, que eu uso. Que eles me dizem quanto eu tenho que ter de pressão*” (HAS/18/C) “*Ah, eu tenho um monte de amigos* [risos]*. Aí fica mandando nesse zap. Tem hora que eu fico: ‘ah, não aguento mais. Só pensa em doença, essa gente’. ‘Para colesterol toma isso, toma aquilo’. Mas eu não tomo nada...*” (HAS/34/F)
Disponibilidade de dispositivos para recebimento de informações, lembretes de agendamentos, entre outros e acesso aos contatos/números de telefone de profissionais/serviços de saúde envolvidos no cuidado	“*Depois que essa profissional se aposentou* [enfermeira]*, eu não tenho um telefone fixo, eu não tenho um e-mail, eu não tenho um WhatsApp, eu não tenho nada que eu fale com qualquer pessoa desse setor*” (PVH/28/B)	“*Porque isso tudo é muito solto: ‘não tá pronto, vai vindo aí* [na UBS buscar informação]*’. Essas coisas soltas assim, me desanimam.* [...] *A gente que paga, gente. Isso me desanima, essa coisa muito solta*” (HAS/30/H)

HAS: usuários com hipertensão arterial sistêmica; PVH: pessoas que vivem com HIV; UBS: unidade básica de saúde.

Fonte: elaboração própria baseada nas entrevistas.

Parte dos usuários com HAS sentia-se à vontade para conversar sobre os problemas de saúde com outros profissionais da EqSF, principalmente naquelas com menor rotatividade. Em relação à comunicação e proximidade, os técnicos de enfermagem destacaram-se como importante apoio para relações e diálogos que abordavam questões para além do agravo. Quando percebiam ausência de empatia, em qualquer tipo de serviço de saúde, os usuários se retraíam. De forma geral, a rotatividade e o pós pandemia de COVID-19 afetaram a construção de relações dialógicas na APS (Quadro 3). Uma fala recorrente sintetizava tal percepção: “*Não sei nem quem tá aqui* [UBS] *agora*” (HAS/02/A).

As PVH mencionaram o grande número de atendimentos como um dos fatores que dificultava a comunicação, pela mitigação do tempo disponível. Poucos não compreendiam com clareza exames, efeitos adversos ou tratamentos recomendados e seguiam irrestritamente as orientações dos profissionais de saúde, que orientavam decisões importantes em suas vidas. Ainda assim, uma participante, travesti, considerou o tempo da consulta curto para o esclarecimento de dúvidas, por exemplo, sobre efeitos adversos relacionados ao uso da terapia antirretroviral (TARV) (Quadro 3).

As ações coletivas voltadas à produção de literacia em saúde e autocuidado nos serviços de saúde estiveram ausentes na fala da quase totalidade dos participantes. Como práticas de autocuidado relacionadas à HAS, pouco mais da metade referiu realizar monitoramento da pressão arterial em domicílio com uso de aparelho digital, sem regularidade. Quanto às PVH, recebiam orientações sobre alimentação e realização de atividades físicas, em frequências distintas entre as policlínicas. Muitas relataram tentar adotá-las, como forma de prevenção de doenças ocasionadas pelo uso contínuo dos antirretrovirais. A maioria tinha fácil acesso a preservativos gratuitos. Foram comuns relatos de associação do uso da TARV como autocuidado para se garantir boa saúde, vida longa, carga viral indetectável e prevenção de transmissão sexual (Quadro 3).

Ainda que parte dos usuários tenha mencionado médicos, técnicos de enfermagem e ACS como profissionais de referência para acompanhamento da HAS, em intensidade distinta, e infectologistas e enfermeiros no caso das PVH, tal relação estava circunscrita aos cuidados clínicos prestados nas UBS e policlínicas. Foram escassos relatos sobre orientações acerca da HAS durante as visitas dos ACS ou por meio de alguma atividade coletiva ou educacional. Em ambos os casos - HAS e PVH - as abordagens individuais ocorriam durante as consultas e eram bastante valorizadas pelos usuários (Quadro 3).

A internet foi relatada por três participantes com HAS, mais jovens, como fonte de informação em saúde. Em divergência, parte deles referiu dificuldades para sua utilização ou falta de confiança nas informações. Quanto às PVH, metade delas utilizava a internet como importante fonte de informação em saúde sobre HIV/aids, com buscas relacionadas à cura, vacinação, novas terapias e pesquisas científicas, preconceito, alimentação, entre outros temas. As demais consideravam o infectologista, televisão, revista, jornal e amigos - alguns, também, vivendo com HIV - como fontes confiáveis. Muitos relataram consultar sites que avaliavam como confiáveis e o infectologista para evitar *fake news*. Por outro lado, dificuldades de acesso a outros profissionais de saúde na RAS, ocasionavam realização de procedimentos sem orientação adequada com base em informações da internet (Quadro 3).

Nenhum participante com HAS ou PVH mencionou estratégias institucionais ou dispositivos para recebimento de informações. No caso dos participantes com diagnóstico de HAS, a informação sobre agendamentos de consultas ou exames exigia a visita domiciliar do ACS ou, em muitos casos, consecutivas idas à UBS (Quadro 3). Os participantes não identificaram canais formais de comunicação (telefone, mensagem ou e-mail) para contato com um profissional ou serviço de saúde de referência. Tal ausência foi motivo de queixa de algumas PVH, pois não recebiam aviso do cancelamento da consulta ou necessitavam reagendá-la presencialmente. Em algumas policlínicas, havia tal contato, realizado informalmente pelo enfermeiro, por meio do WhatsApp pessoal, com interrupção quando ocorria substituição do profissional. O aplicativo era utilizado como canal de comunicação por poucos infectologistas para orientação clínica entre consultas, por iniciativa pessoal.

## Discussão

Os resultados encontrados reforçam a importância das tecnologias relacionais como preditores de resultados em saúde, cotejando-se experiências diversificadas de usuários na APS e na AE com o intuito de captar, em ambos os níveis assistenciais, fatores facilitadores e que dificultam o alcance da continuidade do cuidado. Os resultados sinalizam aspectos que diferenciam e aproximam as distintas experiências. Em comum, junto ao médico da EqSF ou infectologista se estabeleciam as relações de confiança e o protagonismo na condução das trajetórias assistenciais. Entre as diferenças, para os usuários com HAS acompanhados na APS, as relações de vínculo foram cindidas pela rotatividade dos médicos. Na vacância de vínculos com outros profissionais das EqSF, a continuidade e o acesso foram simultaneamente afetados. Entre as PVH, a característica mais marcante foi a forte relação de confiança construída junto ao infectologista, que apresentava maior estabilidade no serviço de saúde. No caso dos dois agravos, os resultados revelaram um cuidado pouco afeito às práticas interprofissionais e às ações coletivas no fortalecimento da continuidade. De toda forma, os resultados do estudo sinalizam que a continuidade relacional importa, e que é altamente valorizada pelos usuários.

O processo saúde-doença tem uma história que não é natural ^25^, cujo compartilhamento com o profissional de saúde faz parte da construção das relações de confiança, que extrapolam os aspectos biológicos ^26^. Na mesma direção, agravos atravessados pelo estigma e preconceito com o HIV, que determinam desigualdades de diversas ordens - de gênero, sociais, entre outras ^27^ - quando compartilhadas com os profissionais podem representar um importante sinal de alerta para a mudanças em suas práticas que, muitas vezes, invisibilizam tais dimensões do processo saúde-doença-cuidado ^28^.

Confiança é um construto psicológico que influencia os comportamentos em saúde relacionados à adesão às orientações, medicamentos prescritos e medidas preventivas ^29^, essenciais no manejo de condições crônicas. Consultas com o mesmo profissional ajudam a desenvolver uma relação de confiança e segurança enquanto ambientes e profissionais desconhecidos podem gerar ansiedade e estresse ^3^ ^,^ ^4^. Embora exista uma confiança institucional *a priori* concedida aos médicos, esta pode ser limitada e enfraquecida por diversos fatores ^3^, como verificado nas experiências dos usuários com HAS, atravessadas pela rotatividade dos profissionais.

Além disso, a continuidade capacita os profissionais de saúde para compreenderem as esperanças, medos e desejos do paciente aprimorando suas habilidades clínicas e pessoais ^3^, como parece sinalizar a experiência das PVH e não observado no caso dos usuários com HAS em função da rotatividade. Na mesma direção, quando um profissional conhece seu paciente, aumenta sua capacidade de perceber necessidades não óbvias ou não expressas verbalmente, de prestar um melhor atendimento em emergências, além de minimizar a repetição da história clínica e de vida a cada novo encontro, aspecto que traz maior segurança e confiança aos usuários ^4^ ^,^ ^30^. A repetição da história clínica a cada novo atendimento, como encontrado na experiência das pessoas com HAS, é causa de grande insatisfação ^31^. Na mesma direção, para garantir a coerência do cuidado e melhorar a experiência do paciente é necessário que as informações clínicas sejam compartilhadas entre os profissionais de saúde em tempo hábil ^32^, o que pode ser postergado pelo registro repetitivo das mesmas informações.

Conforme mencionado, todas as possibilidades de fortalecer as distintas dimensões da continuidade relacional perdem potência diante da rotatividade profissional, sobretudo médica, problema não equacionado na gestão do trabalho no SUS ^33^ e presente nos mais diversos contextos *pari passu* ao crescimento dos serviços de APS ^4^. A rotatividade compromete, além da continuidade, o acesso aos serviços de saúde, ao passo que, pela expectativa do não atendimento, os usuários passam a não mais buscá-los ^34^, como encontrado na experiência das pessoas com HAS neste estudo.

A rotatividade médica dificultava o acompanhamento dos usuários com HAS, ainda que o cuidado permanecesse excessivamente centrado no médico. Outros profissionais, como os técnicos de enfermagem e ACS, tinham papel secundário, limitando-se a intermediar acesso, principalmente o agendamento de consultas e exames, e realizar procedimentos. Nesse sentido, mesmo quando presentes na cena de cuidados, as demais atuações estavam subsumidas à lógica biomédica ^35^.

Não menos relevante, no caso das PVH e com HAS, foi a secundarização do trabalho dos profissionais de enfermagem no compartilhamento dos cuidados clínicos. Enfermeiros em programas de cuidados transicionais reduzem as re-hospitalizações, os custos e melhoram a qualidade de vida de pessoas com agravos crônicos ^36^. Ademais, atuam na coordenação e continuidade do cuidado, planejando a assistência pós-alta, articulando pontos de atenção, realizando atividades de educação em saúde, gerenciando casos complexos, avaliando aspectos clínicos e sociais, e facilitando a navegação dos usuários pelos serviços de saúde ^12^ ^,^ ^14^. Nessa perspectiva, promover tanto cuidado multiprofissional na APS, com o protagonismo da enfermagem ^37^, parece ser um caminho para fortalecer a coordenação do cuidado nas RAS e contribuir para a percepção de continuidade pelos usuários.

O vínculo com profissionais de enfermagem no acolhimento foi altamente valorizado, especialmente entre as PVH e usuários com HAS. Como tecnologia leve de cuidado ^38^, o acolhimento favoreceu a adesão ao tratamento e a sensação de sentir-se cuidado. A abordagem das médicas cubanas foi recordada pela “delicadeza” e abordagem integral, reforçando a importância dessas competências para o vínculo e a continuidade do cuidado ^13^ ^,^ ^39^ e que, portanto, não podem ser secundarizadas.

A literacia em saúde é um processo multidimensional que abrange as habilidades individuais e coletivas para buscar, avaliar e interpretar informações de saúde, visando o autocuidado ou o cuidado de terceiros ^40^. No estudo com PVH, infectologistas centralizavam o manejo de informações cruciais para a saúde dos usuários. Pessoas com HAS também valorizavam a centralidade nos médicos, ainda que os vínculos estivessem esgarçados. Em ambos os grupos, as consultas individuais eram o principal espaço para orientações de saúde, ainda que este espaço seja reconhecido pela assimetria de poder e no qual se deposita no profissional de saúde a expectativa pela resolução dos problemas de saúde ^10^. Práticas coletivas de educação em saúde ^40^, ou mesmo extramuros, durante a visita dos ACS poderiam ser estratégicas para ampliar a literacia em uma perspectiva que incluiria experiências diversas, com compartilhamento de saberes e práticas de cuidado.

A disponibilidade de informações personalizadas é essencial para a continuidade relacional e mantém relação direta com o domínio informacional. De toda forma, a ausência de tecnologias de informação e comunicação (TIC), como encontrado neste estudo, dificulta a prestação de cuidados centrados na pessoa. As TIC têm o potencial de melhorar a qualidade, o compartilhamento de informações e a acessibilidade dos usuários, minimizando, por exemplo, a necessidade de visitas presenciais ^41^.

A continuidade relacional fortalece a segurança e evita a repetição de histórias clínicas, facilitando a navegação no sistema de saúde ^30^. Neste estudo não foram identificadas estratégias formais, como PTS, para viabilizar o contato e compartilhamento de planos terapêuticos entre usuários e profissionais da RAS, predominando as iniciativas informais. Tais aspectos sinalizam, concomitantemente, fragilidades nos domínios da continuidade gerencial e informacional. Nestes casos, embora pacientes e familiares sejam alçados ao posto de único elo entre os distintos pontos de atenção com a missão de facilitar a continuidade assistencial, tal responsabilidade não vem acompanhada das habilidades ou confiança necessárias ^42^.

Os resultados deste estudo ratificam que a continuidade não pode ser analisada assumindo-se que outros atributos dos sistemas e do cuidado em saúde estejam plenamente alcançados. Uma compreensão ampla que possa ser traduzida em políticas, ações e práticas para alcance da continuidade carece de um olhar para o contexto global, especialmente no que diz respeito ao acesso e à qualidade da atenção em saúde ^21^.

Como limites do estudo, destaca-se que a complexidade inerente aos cuidados de pessoas com necessidades complexas requer mecanismos no plano individual que garantam a continuidade relacional, assim como ações no âmbito organizacional do sistema de saúde capazes de mitigar a fragmentação assistencial e que não foram foco deste estudo. A garantia de continuidade informacional e gerencial faz parte deste amplo conjunto de estratégias, não abordadas diretamente neste artigo, ainda que interrelacionadas. De toda forma, argumenta-se que a diversidade de experiências, ancoradas na realidade dos participantes em diferentes sets de cuidado proporcionam uma densa análise da realidade, o que não exclui outras experiências e interpretações.

## Considerações finais

Na interface entre população e sistema de saúde, o profissional deve exercer sua prática orientada à pessoa de modo a tornar possível uma relação de confiança e reciprocidade terapêutica. Os resultados deste artigo sinalizam a importância de um ponto regular de cuidado para a consecução do vínculo e consequente continuidade relacional. Por outro lado, problemas relacionados à gestão do trabalho na saúde, contribui para rotatividade profissional e sequenciais rupturas no seguimento terapêutico com danos para todos - usuários, profissionais e gestores. Argumenta-se ainda que, a continuidade do cuidado precisa ser transversal à RAS e se fortalece na medida em que as equipes multiprofissionais se envolvem na produção de projetos terapêuticos coerentes, coordenados e em sinergia às necessidades e interesses dos usuários. No caso dos agravos crônicos e doenças de curso longo, a perda na continuidade relacional compromete a adesão e a fidelização, levando a reagudizações, ao abandono de tratamento, à desistência de buscar cuidados ou à procura de serviços privados e de pronto-atendimento, ampliando iniquidades entre grupos mais vulnerabilizados.
